# Identification and Field Assay of Two Aggregation Pheromone Components Emitted by Males of the Bark Beetle *Polygraphus punctifrons* (Coleoptera: Curculionidae)

**DOI:** 10.1007/s10886-019-01056-6

**Published:** 2019-02-23

**Authors:** Rizan Rahmani, Erika A. Wallin, Lina Viklund, Martin Schroeder, Erik Hedenström

**Affiliations:** 1 Eco-Chemistry, Department of Chemical Engineering, Mid Sweden University, SE-851 70 Sundsvall, Sweden; 2 Department of Ecology, Swedish University of Agricultural Sciences, Box 7044, SE-750 07 Uppsala, Sweden

**Keywords:** Preparative fraction collection, Enantiomeric separation, SPME, (+)-(1*R*,2*S*)-Grandisol, (−)-(*R*)-Terpinen-4-ol, *Picea abies*

## Abstract

The bark beetle *Polygraphus punctifrons* (Coleoptera: Curculionidae) is a species that feeds on Norway spruce (*Picea abies*) and is found in the Northern parts of Europe and Russia. The release of volatile organic compounds (VOCs) produced by males and females of *P. punctifrons* when the beetles bore into spruce stem sections in a laboratory environment was studied using solid phase microextraction (SPME). The sampled VOCs emitted by boring beetles were analysed by gas chromatography and mass spectrometry (GCMS). (+)-2-[(1*R*,2*S*)-1-Methyl-2-(prop-1-en-2-yl)cyclobutyl]ethanol [(+)-(1*R*,2*S*)-grandisol] and (−)-(*R*)-1-isopropyl-4-methyl-3-cyclohexen-1-ol [(−)-(*R*)-terpinen-4-ol] were identified to be male specific volatiles. The identity of the compounds was confirmed by comparison with synthetic samples. Field trials with synthetic compounds in Sweden showed that racemic grandisol per se was strongly attractive for both males and females, while (−)-(*R*)-terpinen-4-ol was not. Further, when adding (−)-(*R*)-terpinen-4-ol to *rac*-grandisol, a synergistic effect was observed as the trap catch of *P. punctifrons* was fourfold. (−)-(*R*)-Terpinen-4-ol by its own did not attract *P. punctifrons* but *Polygraphus poligraphus,* and the latter was also attracted to traps baited with a 10:90 mixture of the two compounds. Thus, we have identified (+)-(1*R*,2*S*)-grandisol as a main component and (−)-(*R*)-terpinen-4-ol as a minor component of the aggregation pheromone of *P. punctifrons.* This opens future possibilities to monitor and, if necessary, manage populations of *P. punctifrons*.

**Electronic supplementary material:**

The online version of this article (10.1007/s10886-019-01056-6) contains supplementary material, which is available to authorized users.

## Introduction

Besides bark beetle species in the genera *Dendroctonus* and *Ips*, species of other bark beetle genera may cause considerable tree mortality. One example is the genus *Polygraphus*. In North America, *P. rufipennis* can kill black spruce (Bowers et al. 1991), and in Russia, the invasive *P. proximus*, originating from the most eastern part of Russia, have killed large numbers of Siberian fir (Kerchev 2014 and references therein). In Sweden, three *Polygraphus* species occur: *P. poligraphus*, *P. punctifrons,* and *P. subopacus*. *P. poligraphus* is distributed over the whole country, while the other two species occur in more northern parts. All three species reproduce in Norway spruce and are polygamous (one male and several females in the gallery systems). *P. poligraphus* is known to kill spruce trees during warm and dry summers (Lekander 1959; Hande 1979). Central Sweden recently experienced a large bark beetle outbreak, and at least 3 million m^3^ of timber were killed between 2008 and 2011 (Wulff, personal communication). Unexpectedly, not only *I. typographus* but also *P. poligraphus* and to some extent also *P. punctifrons* and *P. subopacus* were present in many of the killed trees (Schroeder and Wulff, personal communication). Thus, it is important to increase the knowledge of the biology of these species.

Tree-killing bark beetles (and their associated microorganisms) attacking living trees are confronted with diverse tree defence mechanisms (Franceschi et al. 2005; Krokene 2015).

Thus, to overcome tree defences, a coordinated mass attack of beetles is necessary (Berryman 1982; Mulock and Christiansen 1986). When bark beetles bore into trees, they produce aggregation pheromones from precursors in the tree or de novo, which coordinates mass attacks of beetles (Blomquist et al. 2010; Tittiger and Blomquist 2016). In *Polygraphus,* it is the male that initiates the attack and produces the aggregation pheromone. So far, pheromones have been identified for *P. poligraphus* (Rahmani et al. 2015; Schurig et al. 1985) and *P. rufipennis* (Bowers et al. 1991)*.*

In the present study, we identified (+)-(1*R*,2*S*)-grandisol, and (−)-(*R*)-terpinen-4-ol as components of the aggregation pheromone of male *P. punctifrons*. The obtained results may have practical applications for the management and monitoring of *P. punctifrons*.

## Methods and Materials

### Insects

The *P. punctifrons* used in pheromone studies in 2015 originated from naturally colonised material collected in the province of Medelpad, Västernorrlands län (Sweden).

The insects used for laboratory experiments in 2018 were also caught at a location in Medelpad. Traps baited with racemic grandisol were used, and insects were collected after four to seven days of trapping and were stored in the refrigerator until they were used.

### VOC Sampling on SPME and GCMS Analysis

Spruce stem sections, with 10 to 15 cm in diameter, were cut one to three weeks before they were used in experiments and during that time stored at 5 °C. Newly emerged *P. punctifrons* males and females were placed on the stem sections in 1.5 ml Eppendorf microtubes (Sarstedt, Nümbercht, Germany), which were nailed on to the stem sections to collect volatiles released from the holes bored by the colonising beetles. In all of the experiments, an SPME fibre (pink or yellow – see below) was placed in the cut end of the microtube, and the gap between the SPME fibre and the opening was sealed with aluminium foil. All experiments were performed in the laboratory during April–July 2015 and August 2018 and at room temperature (20–22 °C). The stem sections were exposed to natural daylight for 6 h between 09:00 and 15:00 and otherwise kept without direct natural daylight. VOC sampling for 1 h using an SPME fibre conditioned for 10 min at 250 °C before sampling, and GCMS analysis was normally performed once per day in each experiment.

In 2015, a total of 45 insects were separately introduced into Eppendorf microtubes nailed on three stems of Norway spruce. Fifteen insects were introduced to each stem, six females and nine males on the first stem (21–23 April), seven females and eight males to the second stem (21–23 April), and for the third stem (2 May) five females and ten males were introduced. In total nine males were followed by GCMS, and five of them produced the pheromone. In 2018, six males were introduced (20 August) to one stem, and three of them produced a pheromone that was analysed by GCMS. For background emission, each stem was manually drilled, and an empty Eppendorf tube was nailed onto the hole. VOCs were collected by using two different types of SPME fibres, 65-μm polydimethylsiloxane/divinylbenzene (PDMS/DVB, Pink 57,326-U) and 30-μm polydimethylsiloxane (PDMS, Yellow 57,309-U) (Supelco, Bellefonte, PA, USA). A Hewlett-Packard 6890 N GC (Agilent Technologies, Santa Clara, CA, USA) using an HP 5973 mass spectrometer (MS) operating in electron impact (EI, 70 eV) ionisation mode for detection was used to analyse the SPME samples. The mobile phase (flow rate = 1.0 mL/min) was helium, the split/splitless injector was operated in splitless mode at 250 °C, the transfer line was maintained at 230 °C, and the auxiliary temperature was set to 200 °C.

Different types of columns were applied including a polar column VF-23 ms (30 m × 0.25 mm i.d. and 0.25 μm film thickness; Agilent J&W Scientific, Folsom, CA, USA), the oven temperature was programmed from 50 °C after 2 min, then at 5 °C/min to 230 °C and held for 10 min. The second column was a mid-polar HP-5 ms column (30 m  ×  0.25 mm i.d., 0.25 μm film thickness; Agilent J&W Scientific, Folsom, CA, USA), the oven temperature was programmed from 50 °C after 2 min, then at 10 °C/min to 230 °C and held for 10 min. In addition, an enantioselective column of BETA DEX™ 225 (30 m × 0.25 mm i.d. and 0.25 μm film thickness, Supelco, Bellefonte, PA, USA) was used with an initial temperature at 50 °C, held for 2 min, then increased to 200 °C at 5 °C/min. Desorption time for the fibre was 5 min.

The raw MS data was analysed using the Chemstation (D.03.00.611) program (Agilent), and compounds were identified by comparing fragmentation patterns observed in the mass spectra with literature data and the NIST MS 2.0 mass spectral libraries with reverse and forward match values. The identities of assigned structures were also secured by comparing retention times and mass spectra of the natural products with those of synthetic references.

### Preparative Enantiomeric Separation and Collection of (+)-(1R,2S)-Grandisol and (−)-(1S,2R)-Grandisol Using a PFC Connected to a GCFID

Preparative enantiomeric GC separation of racemic grandisol was performed on an Agilent 7890A GCFID (Santa Clara, CA, USA) equipped with an enantioselective semi-preparative column (Rt-βDEXsm 30 m × 0.32 mm i.d., 0.25 μm film thickness, Restek, Bellefonte, PA, USA). For the oven temperature program used for separation and collection of the enantiomers, the temperature was initially 100 °C, then increased to 150 °C at 3 °C/min. The gas chromatograph was run with a constant flow of 2 mL/min of helium with a PFC switch temperature of 210 °C, transfer line temperature of 200 °C, and two 1 μL glass fraction collectors. In each run, 1 μL of samples were injected in splitless mode.

Optical rotation measurements of the separated samples were carried out on a Perkin–Elmer 341 Polarimeter (D = 589 nm, Na lamp, 1 dm cell) and are reported in degrees.

### Chemicals

*n*-Hexane (HPLC grade, Sigma-Aldrich, Schnelldorf, Germany) was used for LC purification and *n*-nonane (GC grade, Sigma-Aldrich, Schnelldorf, Germany) as solvent in field trials 2016. Racemic grandisol (Grandlure I) was purchased from Bedoukian Research (Danbury, CT, USA), and (−)-(*R*)-terpinen-4-ol (50% ee) was purchased from TCI (Portland, OR, USA). Enantiomerically pure (−)-(*R*)-terpinen-4-ol (>99% ee) was obtained by converting commercially available (−)-(*R*)-terpinen-4-ol (50% ee) to its 3,5-dinitrobenzoyl derivative, which was recrystallized in ethanol and then in isopropanol. The (−)-(*R*)-enantiomer was first enriched in solution and then in the crystals, and the 3,5-dinitrobenzoate was hydrolysed (full experimental details are presented in the supplementary material).

Frontalin was purchased from Contech (Delta, BC, Canada) and Synergy Semiochemicals (Burnaby, BC, Canada).

**Scheme 1:**
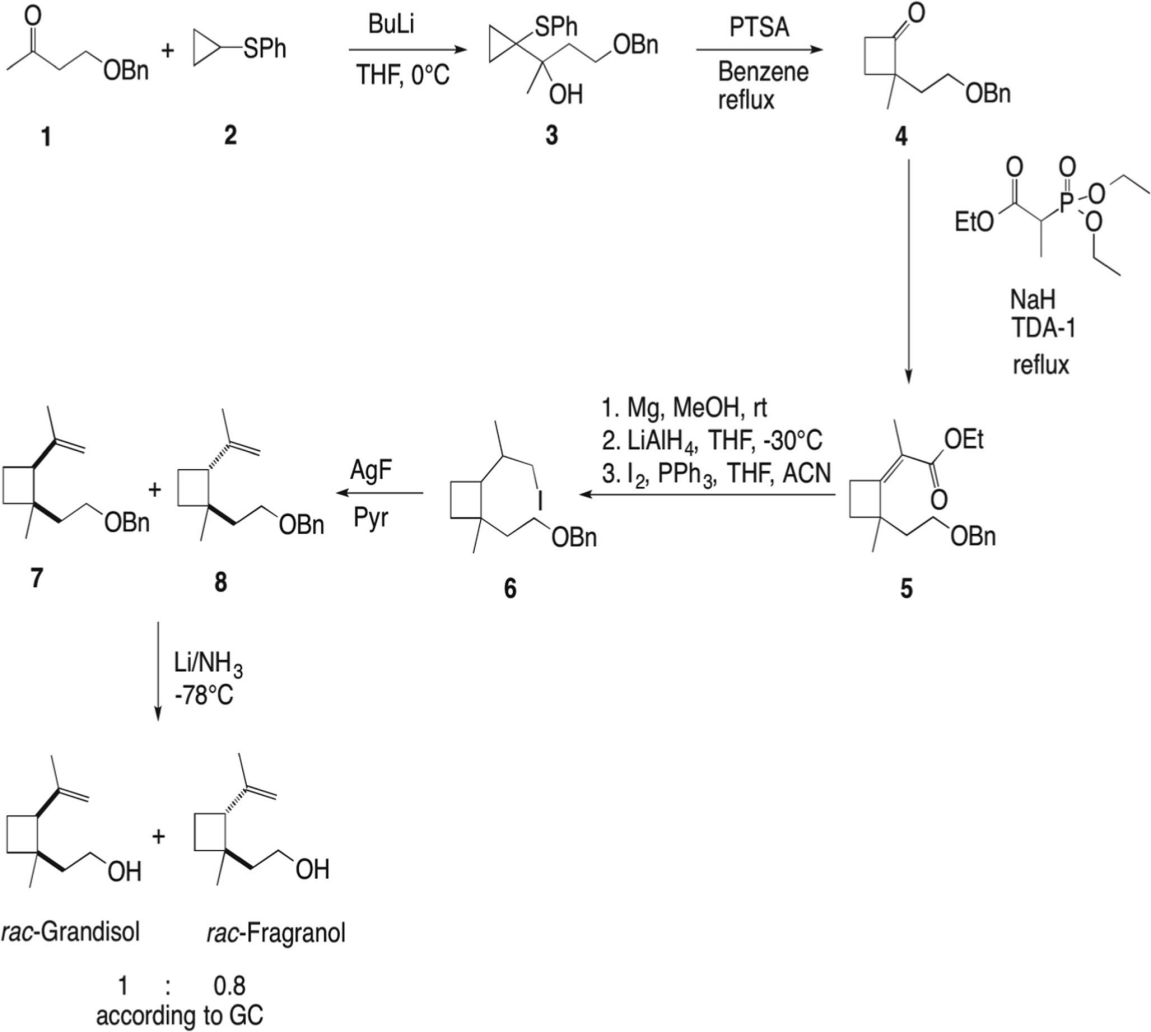
The synthetic pathway to produce a 1:0.8 mixture of racemic grandisol and racemic fragranol

A mixture of racemic grandisol and racemic fragranol was synthesised from cyclopropylphenylsulfide and 4-(benzyloxy)butane-2-one according to the method of Bernard et al. 2003 (Scheme 1). Full experimental details are presented in the supplementary material.

For field studies, racemic grandisol and enantiomerically pure (−)-(*R*)-terpinen-4-ol (> 99% ee) were used. In 2015, the average release rate of grandisol from the dispensers was 12 μg/day. This was determined as an average weight loss during 16 weeks in a well ventilated laboratory at 20–22 °C. In 2016, the average release rates from the dispensers were 0.29 mg/day for grandisol, 0.038 mg/day for terpinen-4-ol, and 0.38 mg/day for frontalin. This was determined as an average weight loss during the field experiment. The release rate of (−)-(*R*)-terpinen-4-ol was approximately 10% of the release rate of racemic grandisol to approximate the proportions seen in the insect, which was roughly estimated by comparing GC peak areas from insect samples.

### Field Tests

Two field experiments were conducted in Sweden in 2015 and 2016 in the province of Medelpad, Västernorrlands län (Sweden). Black Ecotraps (Fytofarm Ltd.) with collection jars for dry catches were used. The experiments were conducted in spruce dominated forests at 10 separate locations for each year. Three of the locations were used in both 2015 and 2016, while the other seven locations were changed. Traps were spaced at least 30 m apart from each other. In 2015, traps were baited July 7–9 and emptied weekly until September 29–30. In 2016, traps were baited on June 21–23 and were emptied every week until August 30–31. However, due to large catches in 2016, only catches from the first week were counted and included in the analysis.

The experiment in 2015 included two treatments at ten locations: (1) 100 mg of racemic grandisol in a 400 μl polyethylene dispenser (Synergy Semiochemicals Corp.) and an empty dispenser as a control. Racemic grandisol was chosen for the experiment as it is commercially available.

In 2016, the field experiment included six treatments at ten locations: (1) 50 mg of racemic grandisol, (2) 50 mg of racemic frontalin, (3) 5 mg of (−)-(*R*)-terpinen-4-ol (> 99% ee), (4) 50 mg of racemic grandisol with 50 mg of racemic frontalin, (5) 50 mg of racemic grandisol with 5 mg of (−)-(*R*)-terpinen-4-ol and (6) a control. (−)-(*R*)-Terpinen-4-ol was prepared in our laboratory and included in the field experiment because it is quantitatively a minor compound emitted by *P. punctifrons* males. Frontalin has been shown to increase the catches of *P. poligraphus* when added to the species’ pheromone (Kohnle et al. 1985). Slightly modified “wick baits” were used as dispensers (Birgersson et al. 2012). The compound(s) for each bait was dissolved in 8 mL of *n*-nonane and placed in a 12 mL glass vial. The dissolved compound(s) were allowed to evaporate through a Teflon tube, 8 cm × 1.5 mm i.d. which was lined with cotton yarn and inserted through a drilled hole in the lid of the vial.

In both years, the field studies were part of a larger study focused on evaluating attractants and repellants for *P. poligraphus*. Dispensers were weighed before and after the experiment in 2016 to estimate release rates, whereas in 2015 release rates were measured as an average weight loss during 16 weeks in a well ventilated laboratory at 20–22 °C. Collected insects were stored in the freezer until identification could be done. Beetles were identified and sexed in the laboratory using a stereomicroscope (Sagitta) with 14–90 times magnification (Lekander 1959). In 2015, all insects were identified, but in 2016, due to the large number of *Polygraphus* caught and the time-consuming determination of *Polygraphus* species, a sample of 50 beetles per trap was investigated.

### Statistial Analyses

Treatment effects were compared by analyzing the number of caught beetles, using a two-sided Student’s T test assuming unequal variances. A paired test was used since the population size was expected to vary in the different locations. Each location was considered a replication and the significance level was set to α = 0.05. Statistical tests were performed in Microsoft Excel using built in functions. The average number of caught beetles per trap with a 95% confidence interval was also calculated.

## Results and Discussion

### VOC Sampling on SPME and GCMS Analysis

**Fig. 1:**
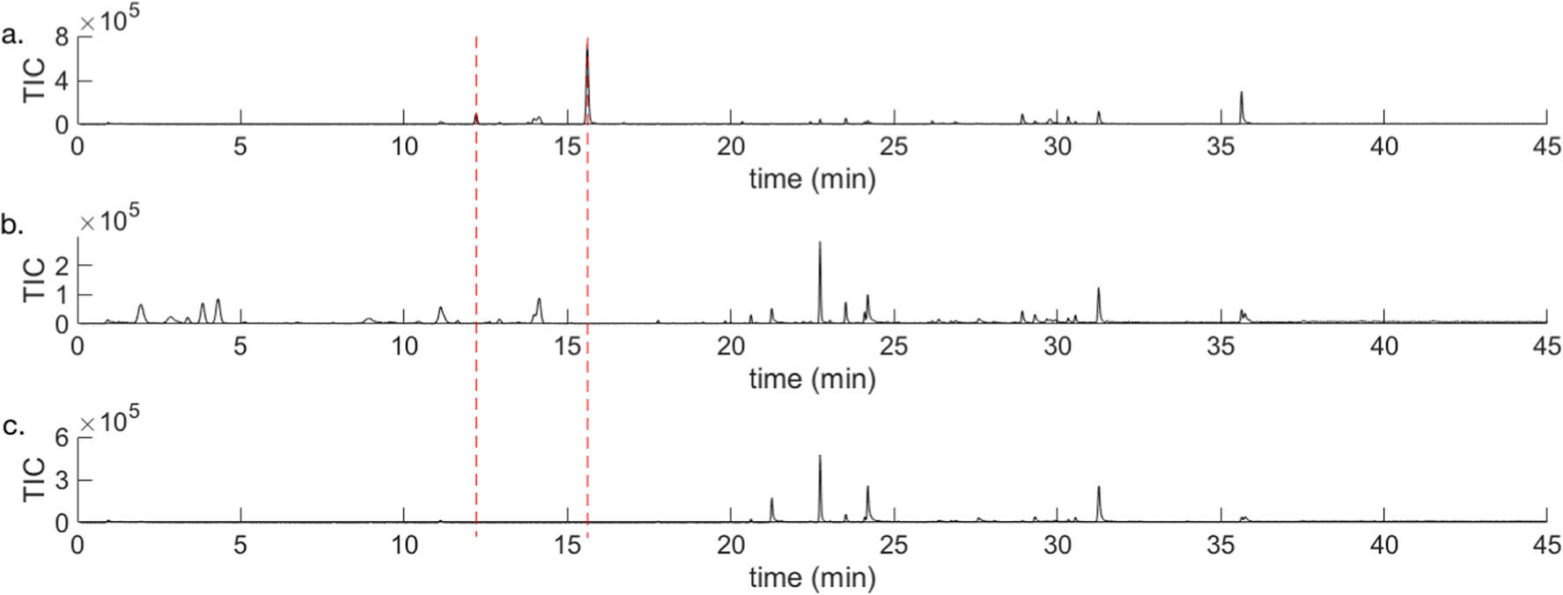
GCMS analysis using a VF-23 ms column. GC/MS chromatogram of VOCs sampled by SPME (yellow fibre) **a**) from a representative boring male of *P*. *punctifrons* on a spruce stem section, **b** from a spruce stem with a representative boring female of *P*. *punctifrons*, **c** from a representative spruce stem that has been manually disturbed by drilling. The dashed red lines show that the compound at 12.20 min and the compound at 15.61 min are not found in the female and the background emission

This work reports the characterisation of volatile chemicals emitted by *P. punctifrons* males and the identification of two compounds emitted by boring males. Five of the boring males that were followed by GCMS produced one compound in high amounts at retention time 15.61 min (Fig. 1a). The compound was absent in the spruce log (Fig. 1b) and not released by females (Fig. 1c). This interesting discovery prompted us to have a closer look at the identity of this male-specific compound and also on a smaller peak emitted by males at retention time 12.20 min. NIST MS search 2.0 library and reverse and forward match values were used to extract the best hits when comparing observed fragmentation patterns in the mass spectra of the unidentified compounds. For the peak eluting at 15.61 min the two most promising hits were suggested as grandisol (with a forward match value of 955 and a reverse match value of 955) and fragranol (with a forward match value of 814 and a reverse match value of 921). Consequently, the identity for the compound emitted in high amounts was nearly equally probabable for the two diastereomeric monoterpenes grandisol and fragranol.

For the male-specific compound at retention time 12.20 min forward match values of 919 and reverse match 919 was obtained, and the highest rated hit correlated to 1-isopropyl-4-methyl-3-cyclohexen-1-ol resp. *p*-menth-1-en-4-ol, which is terpinen-4-ol.

**Fig. 2:**
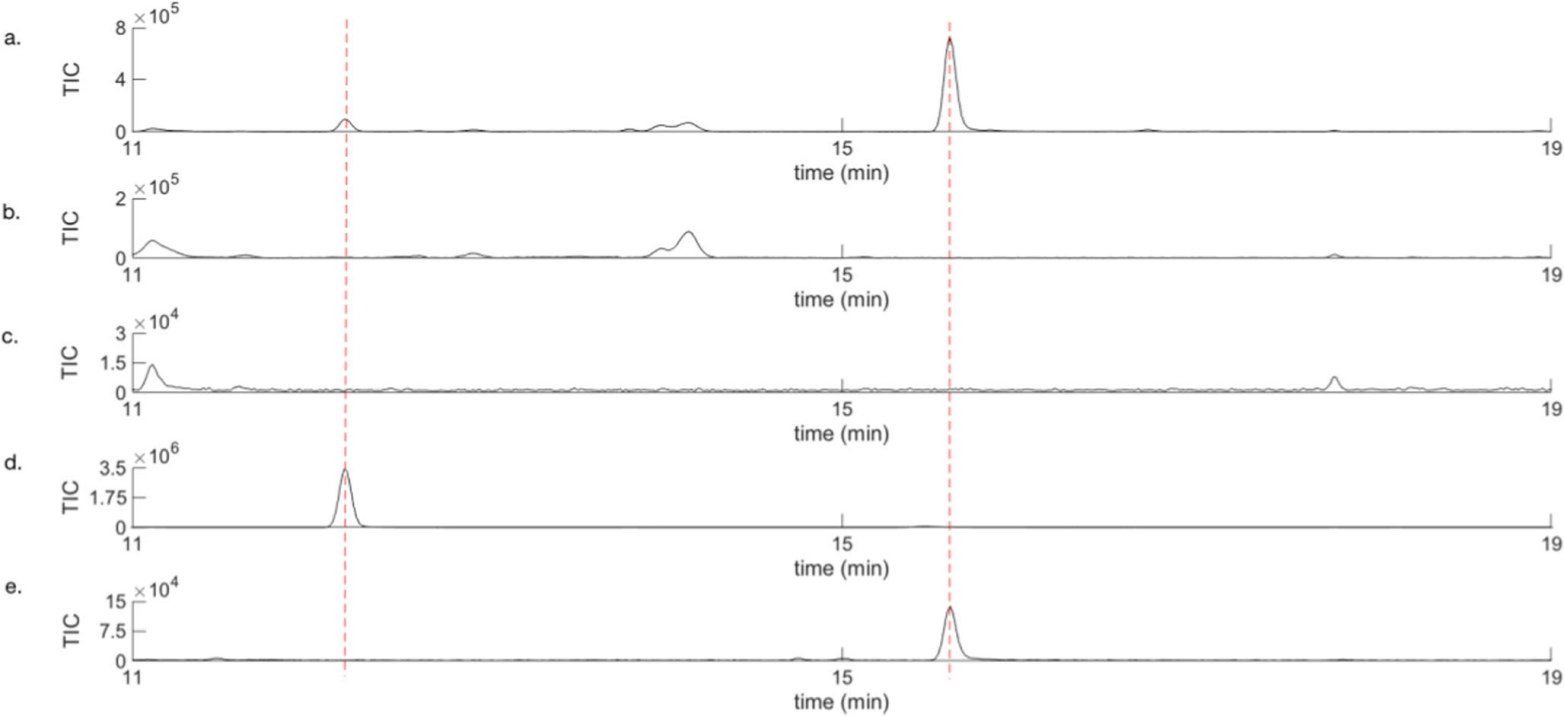
GCMS analysis using a VF-23 ms column. Zoomed in parts of GCMS chromatograms of **a**) VOCs from a representative boring male of *P*. *punctifrons* on a spruce stem section sampled by SPME (yellow fibre), **b** VOCs from a spruce stem with a representative boring female of *P*. *punctifrons* sampled by SPME (yellow fibre)*,***c** VOCs from a representative spruce stem that has been manually disturbed by drilling and then sampled by SPME (yellow fibre), **d** synthetic reference of terpinen-4-ol, **e** synthetic reference of Grandlure I (grandisol)

The identities of the two compounds were further supported, when performing GCMS analysis using both an HP-5 ms (not shown) and VF-23 ms column and comparing retention times for the two unknown volatiles (Fig. 2) with synthetic reference compounds. Overlap was obtained with terpinen-4-ol and Grandlure I (grandisol) respectively.

### Synthesis

The very similar mass spectra of grandisol and fragranol prompted us to synthesise a reference mixture containing both diastereomers in a known ratio according to an established reaction pathway (Bernard et al. 2003). The synthesis started (Scheme 1) from 4-(benzyloxy)butan-2-one (**1**) obtained via the reaction between deprotonated cyclopropyl phenyl sulphide (**2**) resulting in 4-(benzyloxy)-2-(1-(phenylthio)cyclopropyl)butan-2-ol (**3**). 2-(2-(Benzyloxy)ethyl)-2-methylcyclobutan-1-one (**4**) was obtained after a ring expansion under acidic conditions (PTSA in benzene). Compound (**4**) underwent a Wittig-Horner reaction with triethyl phosphonopropionate (60% NaH in oil, TDA-1, THF and reflux) yielding ethyl (*Z*)-2-(2-(2-(benzyloxy)ethyl)-2-methylcyclobutylidene)propanoate (**5**). Two subsequent reductions were performed; reduction of the conjugated double bond using Mg(s) in methanol and reduction of the ester with lithium aluminumhydride in THF. The obtained 2-(2-(2-(benzyloxy)ethyl)-2-methylcyclobutyl)propan-1-ol was transformed to the iodide (**6**) (I_2_, PPh_3_, Imidazole, ACN) followed by dehydroiodination with silver fluoride in dry pyridine, yielding a 1:0.8 diastereomeric mixture (*cis:trans*) of ((2-(1-methyl-2-(prop-1-en-2-yl)cyclobutyl)ethoxy)methyl)benzene (**7**, **8**). The final step was deprotection of the ((2-(1-methyl-2-(prop-1-en-2-yl)cyclobutyl)ethoxy)methyl)benzene performed with Li/NH_3_ resulting in a 1:0.8 mix of grandisol and fragranol (as determined by GC). Column chromatography on silica resulted in a partial separation of the isomers, and three fractions were isolated. One fraction containing only grandisol, one small fraction with only fragranol and one fraction with a mixture of the isomers.

**Fig. 3:**
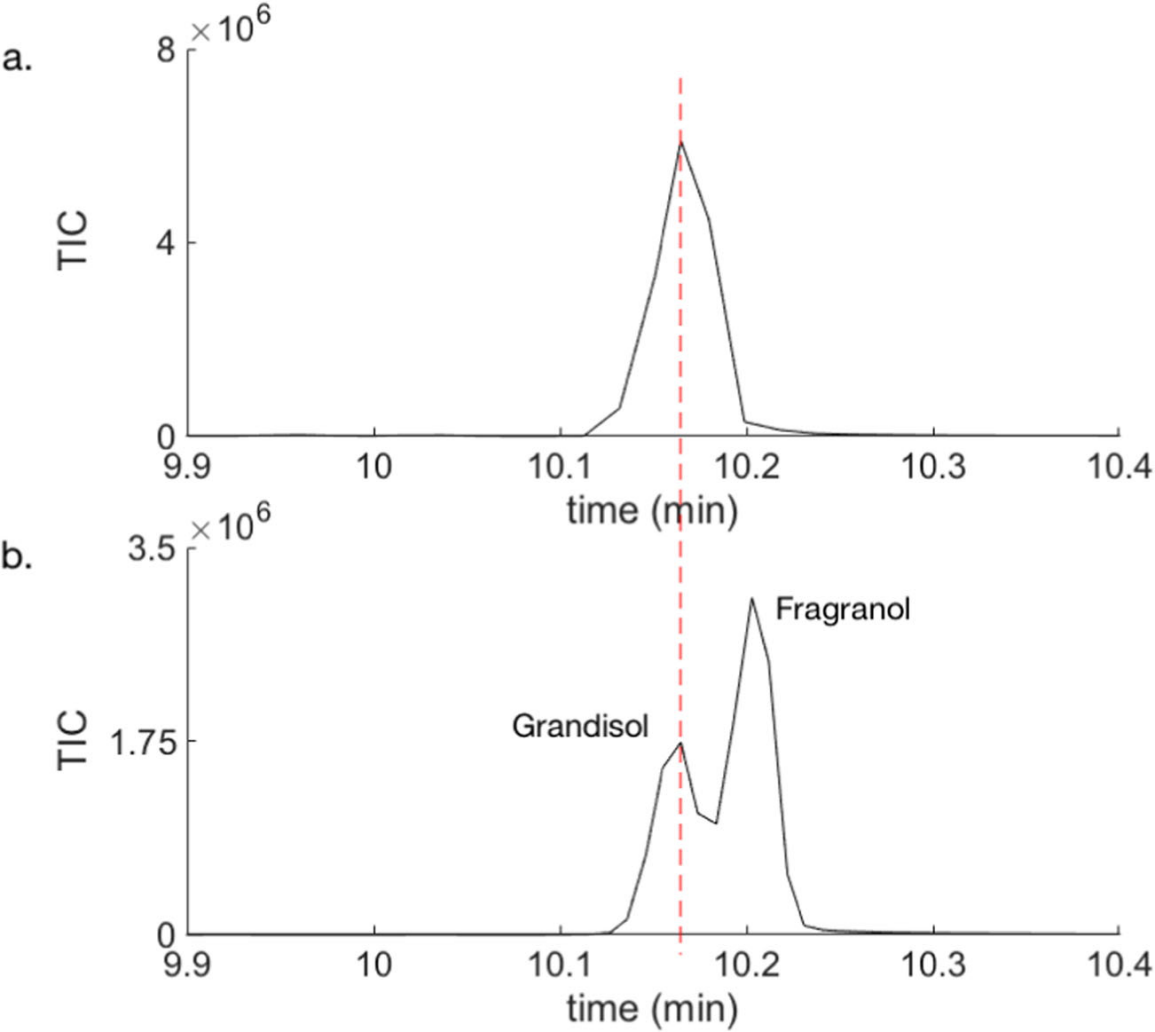
Retention time for grandisol and fragranol on an HP-5 ms column. GCMS analysis of **a**) VOCs from a boring male of *P*. *punctifrons* on a spruce stem section sampled by SPME (pink fibre) and **b**) synthetic mixture after SPE of grandisol (r.t = 10.16 min) and fragranol (r.t = 10.20 min)

The synthesised mixture of diastereomers obtained after SPE was then analysed by GCMS on an HP-5 ms column (Fig. 3) which separated grandisol and fragranol better than the VF-23 ms column.

**Fig. 4:**
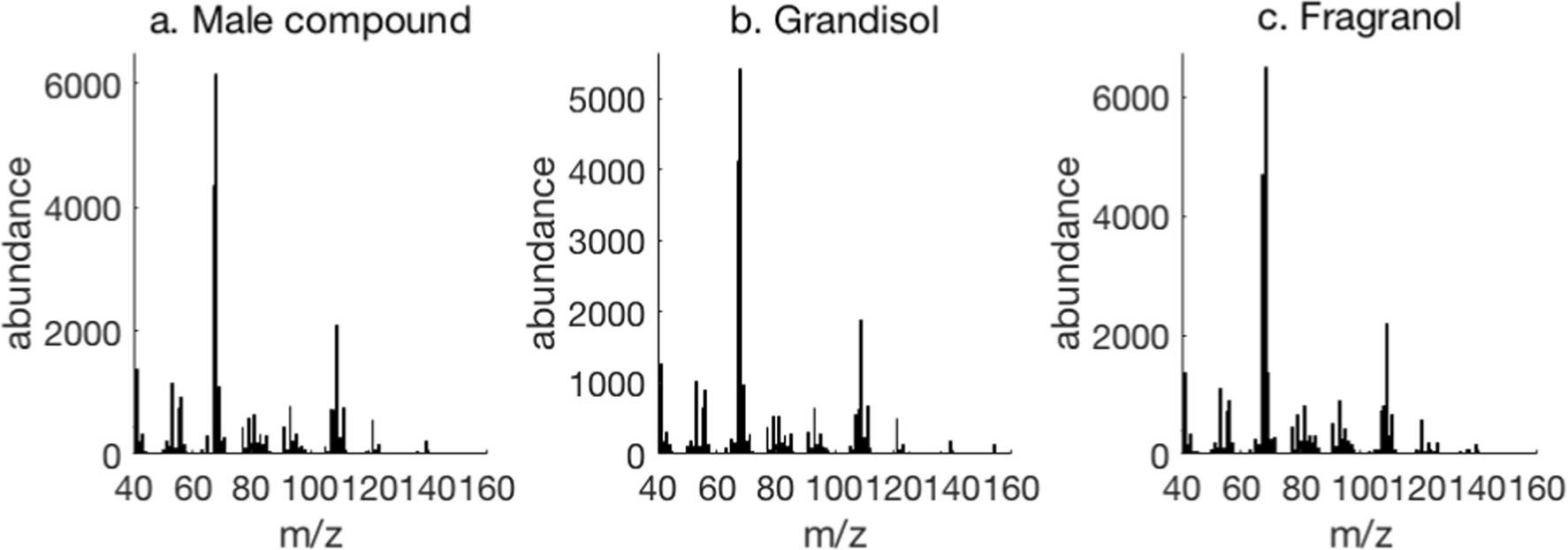
Mass spectra for **a**) the peak from a representative *P*. *punctifrons* male, **b** synthetic grandisol, and **c**) synthetic fragranol

The analysis presented in Fig. 3 conclusively confirmed the male specific compound to be grandisol as the retention times are the same as for grandisol in the synthetic mixture. When we compared the mass spectra from the male emitted compound, synthetic grandisol and fragranol, the spectra were almost identical (Fig. 4).

From our results above, we propose that males of *P. punctifrons* emit grandisol and terpinen-4-ol as a part of an aggregation pheromone. Grandisol was first identified in the cotton boll weevil, *Anthonomus grandis* (Gueldner et al. 1971) and since then also in several other beetle species but only once in a bark beetle, *Pityophthorus pityographus* (Francke et al. 1987). When sampling with SPME fibres (PDMS/DVB) the ratio of grandisol to terpinen-4-ol varied from 99:1 to 80:20 when comparing GC peak areas from the GCMS analysis of volatiles of different males.

### Enantiomeric Analysis of Male Emitted Grandisol and Terpinen-4-ol

**Fig. 5:**
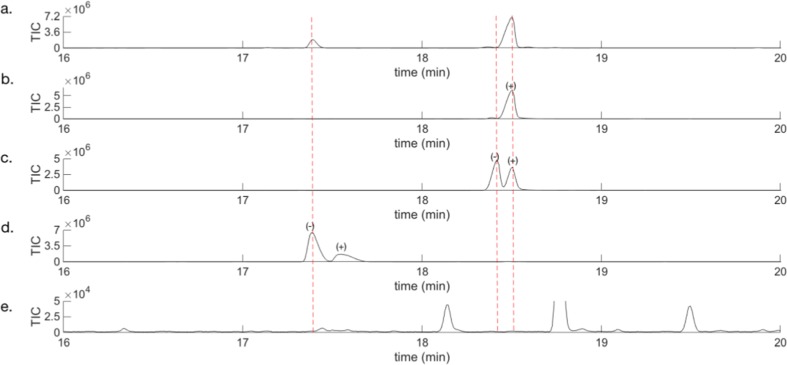
Zoomed in part of chromatograms from the GCMS analysis on a chiral BETA DEX™ 225 column. **a** VOCs released by a boring male of *P*. *punctifrons* on a spruce stem section sampled by SPME (pink fibre), **b** enantiomerically pure (+)-(1*R*,2*S*)-grandisol from PFC trap 1*,***c** enriched (−)-(1*S*,2*R*)-grandisol from PFC trap 2, **d** commercial (−)-(*R*)-terpinen-4-ol showing 50% ee, **e** VOCs from a representative spruce stem that has been affected by manually drilling (sampled by SPME, pink fibre)

It remained to determine the enantiomeric composition of terpinen-4-ol and grandisol from the volatiles produced by male *P. punctifrons*. Earlier work by Francke and Vité (1983), Schurig et al. (1985), and Rahmani et al. (2015) showed that males of *P. poligraphus* emit (−)-(*R*)-terpinen-4-ol of high optical purity. The enantioselective analysis of the production of specific compounds from three *P. punctifrons* males revealed that also in this species only the (−)-(*R*)-terpinen-4-ol enantiomer is produced (Fig. 5a and d).

**Fig. 6:**
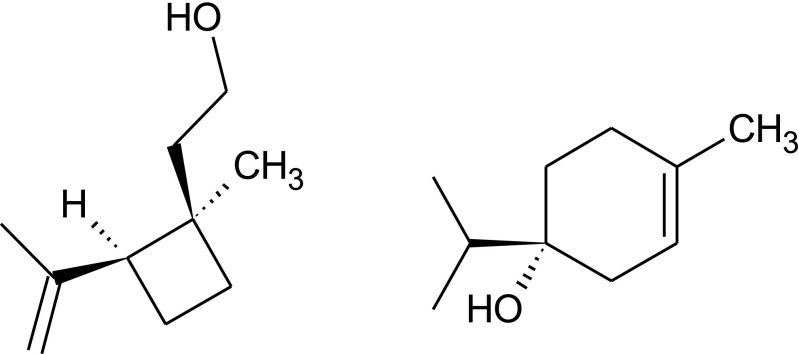
(+)-(1*R*,2*S*)-Grandisol(−)-(*R*)-Terpinen-4-ol

To investigate which enantiomer of grandisol males of *P*. *punctifrons* were emitting, pure synthetic enantiomers were needed for comparison of GC retention times. Instead of a complicated synthesis with many reaction steps and very low yield (Alibés et al. 1996), an alternative and less time-consuming method was chosen: preparative gas chromatography (see, for example, Ravid et al. 1992; Kim et al. 2014). We decided to use a semi-preparative enantioselective GC column, Rt-βDEXsm, to separate the grandisol enantiomers and then collect the pure enantiomers with a fraction collector (PFC). This semi-preparative column showed a different selectivity for the two compounds compared to the analytical BETA DEX™ 225 column, resulting in a reversed elution order of the two enantiomers. Two experiments were run consisting of more than 500 GC injections during two weeks, and two fractions were collected from each experiment and their optical rotation [α]_D_^20^ were measured in *n*-hexane at 20 °C. Experiment one gave +0.2 for fraction one and -0.5 for fraction two, while experiment two gave +0.3 for fraction one and -0.6 for fraction two. The optical rotation sign in combination with enantioselective GC was then used to assign the absolute configuration to the male produced natural grandisol. In Fig. 5a–c it is shown that the enantiomer with the optical rotation sign of (+) is the one produced by males of *P*. *punctifrons*. The enantiomeric purity of the (+)-PFC-fractions was 95.5%, and the (−)-PFC-fractions was 25% ee. Thus, one major component (+)-(1*R*,2*S*)-grandisol (Fig. 6) and one minor component (−)-(*R*)-terpinen-4-ol (Fig. 6) have been detected in the SPME sampled volatiles of adult male *P. punctifrons*.

### Field Study

During the summer of 2015, 643 *P. punctifrons* were caught in the *rac*-grandisol baited traps at 10 locations in the province of Medelpad, and 61% of them were females (Table 1). Only 4 *P. punctifrons* were caught in the unbaited traps. The *rac*-grandisol baited traps caught significantly more beetles than the unbaited traps, both males (Student’s T test, paired, two-tailed, *P* = 0.03) and females (*P* = 0.04).

**Table 1:** Trap catches of *P. punctifrons*, July 7–September 30, 2015. Catches are presented as a total of males and females from the entire trapping period, and as a mean per trap with a 95% confidence interval (95% CI). There were 10 replications in the experiment (*N* = 10)

Treatment	*P. punctifrons* female		*P.punctifrons* male	
	Total	Mean per trap (95% CI), *N* = 10	Total	Mean per trap (95% CI), *N* = 10
*rac*-Grandisol	392	39 (±36)	251	25 (±22)
Control	3	0 (±1)	1	0 (±0)

In 2016, 16,244 *Polygraphus* beetles were caught in total during one week at 10 locations (Table 2). Beetles were counted, and a subsample of 50 beetles per trap was identified, that is 500 beetles per treatment. The percentage of each species in the subsamples was used to estimate the average number of *P. punctifrons* and *P. poligraphus* caught per trap. For traps baited with *rac*-grandisol as well as traps baited with *rac*-grandisol and frontalin, all beetles in the subsamples were *P. punctifrons*. It was confirmed once again that *rac*-grandisol baited traps caught more beetles than the control (Student’s T test, paired, two-tailed, *P* = 0.001). Traps baited with a mixture of *rac*-grandisol and frontalin did not catch significantly different numbers of beetles than traps baited with only *rac*-grandisol (Student’s T test, paired, two-tailed, *P* = 0.61). For (−)-(*R*)-terpinen-4-ol, all beetles in the subsamples were *P. poligraphus*. As expected, *P. poligraphus* was more attracted to its pheromone (−)-(*R*)-terpinen-4-ol than to the control (Student’s T test, paired, two-tailed, P = 0,01). Traps baited with a mixture of *rac*-grandisol and (−)-(*R*)-terpinen-4-ol caught significantly more *P. punctifrons* than *rac*-grandisol baited traps (Student’s T test, paired, two-tailed, *P* = 0.006) and possibly more *P. poligraphus* than (−)-(*R*)-terpinen-4-ol-baited traps, however, in the last case, the difference was not statistically significant (Student’s T test, paired, two-tailed, *P* = 0.051). The unbaited traps did not catch any beetles, and the frontalin baited traps caught only two beetles in total, both were *P. poligraphus.*

**Table 2:** Trap catches of *Polygraphus* beetles, June 21–June 29, 2016. Catches are presented as a total and as a mean per trap for each species with a 95% confidence interval (95% CI). Estimated numbers are based on the proportion of each species in subsamples of identified beetles. There were 10 replications in the experiment (N = 10)

Treatment	*Polygraphus spp.*	Percentage identified	Estimated number of *P. punctifrons*	Estimated number of *P. poligraphus*
	Total		Mean per trap (95% CI), *N* = 10	Mean per trap (95% CI), *N* = 10
*rac*-Grandisol	2489	20%	249 (±125)	0
Frontalin	2	100%	0	0 (±0)
(−)-(*R*)-Terpinen-4-ol	496	100%	0	50 (±35)
*rac*-Grandisol	2300	21%	230 (±105)	0
& frontalin				
*rac*-Grandisol & (−*)*-(*R*)-terpinen-4-ol	10,957	4.8%	950 (±414)	146 (±122)
Control	0	–	0	0

The large catches in 2016 were probably due to the higher release rate of racemic grandisol from the dispensers as compared to the release rates in 2015. This was possible due to the change of dispenser type, from polyethylene dispensers to “wick baits”. It is likely that the populations of *P. punctifrons* varies from year to year, during the summer and between different sites, which may also partly explain the large difference in trap catches between 2015 and 2016.

Frontalin has been shown to increase catches of *P. poligraphus* when combined with its pheromone terpinen-4-ol (Kohnle et al. 1985), but no such effect could be seen for *P. punctifrons* in this study. However, the combination of *rac*-grandisol and (−)-(*R*)-terpinen-4-ol seems to result in a synergistic effect when it comes to trap catches of *P. punctifrons*.

(−)-(*R*)-Terpinen-4-ol is also the pheromone of *P. poligraphus* (Francke and Vité 1983; Rahmani et al. 2015; Schurig et al. 1985), and it was shown that this species was also caught in the traps baited with *rac*-grandisol and (−)-(*R*)-terpinen-4-ol. The combination may be more attractive to *P. poligraphus* than (−)-(*R*)-terpinen-4-ol alone, but the difference was not statistically significant. Racemic grandisol alone did not attract *P. poligraphus*, based on our subsamples. These somewhat puzzling results indicate that *rac*-grandisol is not a repellent for *P. poligraphus* as might have been expected to differentiate the aggregation pheromones of the two species. A possibility is a missing component in the *P. punctifrons* aggregation pheromone that acts as a repellent for *P. poligraphus* or that a yet unknown compound is necessary to complete the aggregation pheromone of *P. poligraphus* (Francke and Vité 1983). Nevertheless, racemic grandisol may have a similar behavior modifying effect on this species as frontalin (Kohnle et al. 1985). Anyway, this interesting result is not the focus of this work but needs to be investigated further in the future.

### Conclusion

This report presents the first identification of two components in the male produced aggregation pheromone of *P. punctifrons.* One major component (+)-(1*R*,2*S*)-grandisol and one minor component (−)-(*R*)-terpinen-4-ol have been detected in the SPME sampled volatiles of adult male *P. punctifrons*. The compounds were neither detected among the volatiles emitted by adult females nor in the background emission of Norway spruce logs. The two male-specific compounds were identified by comparison with synthetic standards using GCMS. The stereoisomeric composition of the natural products was assigned by using enantioselective GC. Field trials with synthetic compounds showed that racemic grandisol per se was strongly attractive to both males and females. Moreover, when adding (−)-(*R*)-terpinen-4-ol to *rac*-grandisol in a ratio close to what we found when analysing the SPME fibres, a synergistic effect was observed as the trapcatch was fourfold. Our results may have practical utility for future management and monitoring of *P. punctifrons*.

## Electronic supplementary material

ESM 1 (DOCX 46 kb)
