# Supplementary material for: Identification and Field Assay of Two Aggregation Pheromone Components Emitted by Males of the Bark Beetle Polygraphus punctifrons (Coleoptera: Curculionidae)
Source: J Chem Ecol. 2019 Feb 23;45(4):356–65. doi: 10.1007/s10886-019-01056-6 (PMC6477006; doi:10.1007/s10886-019-01056-6)
Supplement: Supplementary file 1 — (DOCX 46 kb) [file 10886_2019_1056_MOESM1_ESM.docx]

Supplementary Material

Identification and field assay of two aggregation pheromone components emitted by males of the bark beetle *PolygraPhus punctifrons*

`

RIZAN RAHMANI,^a^ ERIKA A. WALLIN,^a^ Lina viklund,^a^ MARTIN SCHROEDER^b^ and Erik Hedenström^a^*

^a^Eco-Chemistry, Department of Chemical Engineering, Mid Sweden University, SE-851 70 Sundsvall, Sweden
^b^Department of Ecology, Swedish University of Agricultural Sciences, Box 7044, SE-750 07 Uppsala, Sweden

Synthesis

**General information.** All reactions were performed under Ar(g) atmosphere unless otherwise stated. Dry THF, Et_2_O and ACN was obtained from a solvent purification system (Activated alumina columns, Pure Solv PS-MD-5, Innovative technology, Newburyport, USA) and used in the reactions when dry condition was needed. NaH (60 % in mineral oil) was washed with several portions of pentane then dried under Ar (g) prior to use, according to the standard procedure. Mg (s) and pyridine was purified according to standard methods prior to use. GCFID analysis was performed on a Varian 3300 instrument EC-5 (30m × 0.25 mm × 0.25 um), N_2_ as carrier gas with a flow of 1mL/min, inlet temperature of 250 °C and a detector temperature of 260 °C with split ratio of 20:1. Two different temperature programs were utilized depending on the volatility of the compounds. Initial temperature 70 °C hold for 2 min, then a 10 °C/min increase to 250 °C hold for 5 min or initial temperature 100 °C hold for 2 min, then a 10 °C/min increase to 300 °C hold for 3 min. Enantioselective GCFID analysis was performed on an Agilent 7890 with a chiral BETA DEX™ 225 column (30m × 0.25 mm × 0.25 um). N_2_ was utilized as carrier gas with a flow of 1 mL/min and a split ratio of 20:1. The inlet temperature was set to 250 °C and the detector temperature to 300 °C. Initial temperature 50 °C hold for 2 min, then increase by 1 °C/min until 110 °C, then 8 °C/min until 160 °C. A Hewlett-Packard 6890N gas chromatograph (Waldbronn, Germany) equipped with a polar FactorFOUR VF-23ms column (30 m x 0.25 mm i.d., 0.25 µm film thickness; Varian, Palo Alto, CA, USA) coupled to a HP 5973 mass spectrometer in full scan mode was utilized for GCMS analysis. The injector was operated in splitless mode at 250 °C, and a helium flow rate of 1 ml/min and a transfer line temperature of 280 °C were used. The source temperature was 250 °C with a filament bias voltage of −70 eV. The mass range used was m/z 40 − 400. For each injection 1 µl of sample was used. Flash chromatography was performed on straight-phase silica gel (Merck 60, 230–400 mesh, 0.040–0.063 mm, 10–50 g/g of product mixture) employing a gradient technique with an increasing concentration (0–100%) of either EtOAc in cyclohexane, Et_2_O in *n*-pentane or Et_2_O in petroleum ether. Solid phase extraction (SPE) was performed on the final product utilizing a gradient of EtOAc in pentane (0-15 %, in steps of 1 %). Thin-layer chromatography (TLC) was performed to monitor the progress of the reaction on silica gel plates (Merck 60, precoated aluminium foil) using ethyl acetate in cyclohexane (40:60, v/v) as an eluent; plates were developed by means of ultraviolet irradiation and/or by spraying with vanillin in sulphuric acid and heating at 120 °C. ^1^H- and ^13^C-NMR spectra was run on a Bruker Avance 500 (Karlsruhe, Germany) (^1^H, 500 MHz; ^13^C, 125.8 MHz) spectrometer using deuterated chloroform (CDCl_3_) as solvent and as internal standard. Chemical shifts (δ) are expressed in ppm and coupling constants (*J*) in Hz. Data are reported as follows: chemical shift, multiplicity (s, singlet; d, doublet; t, triplet; q, quartet; br, broad; m, multiplet).

**4-(Benzyloxy)-2-(1-(phenylthio)cyclopropyl)butan-2-ol.** nBuLi (20.6 mL, 1.6M) was slowly added to a stirred solution of cyclopropylphenylsulfide (5 g, 33 mmol) in THF (85 mL) at 0 °C. The resulting mixture was allowed to reach RT and stirred for 5h. 4-(Benzyloxy)butan-2-one (5.88 g, 33 mmol) was added at 0 °C, and the reaction was allowed to reach RT and was stirred over night (17 h). Brine (100 mL) and Et_2_O (50 mL) was added, and the layers were separated. The aqueous phase was extracted with Et_2_O (3×50 mL). The combined organic layers were dried over Na_2_SO_4_(anhydr.). Purification with flash chromatography (EtOAc in cyclohexane) resulted in 2.147 g clear oil (approx. 20 % yield, 88 % chemical purity according to GC analysis). ^1^H-NMR(CDCl_3_): δ 0.91 (m, 4H), 1.26 (s, 3H), 1.97-2.03 (m, 2H), 2.20-2.25 (m, 2H), 3.63-3.67 (m, 1H), 3.76-3.80 (m, 1H), 3.78 (s, 1H), 4.48 (s, 2H), 7.15-7.45 (m, 10H) ppm. Similar to Bernard et al 2003.

**2-(2-(Benzyloxy)ethyl)-2-methylcyclobutan-1-one.** Cyclopropylcarbinol (2.147 g, 6.45 mmol) was mixed with PTSA (1.24 g, 6.54 mmol) in water saturated benzene (40 mL) and refluxed for 4h. The reaction mixture was allowed to reach RT and was subsequently washed with Na_2_CO_3_ (sat.aq., 2×20 mL) and brine (20 mL) and dried over Na_2_SO_4_(anhydr.). Purification via flash chromatography (EtOAc in cyclohexane) resulted a in 0.519 g orange/red oil (36% yield, 80% chemical purity according to GC). ^1^H-NMR(CDCl_3_): δ 1.21 (s, 3H), 1.72-1.82 (m, 2H), 1.94-1.99 (m, 1H), 2.04-2.08 (m, 1H), 3.00 (m, 2H), 3.58 (t, 2H, *J* = 6.5 Hz), 4.47 (q, 2H, *J* = 12 Hz), 7.27-7.36 (m, 5H) ppm. Similar to Bernard et al 2003.

**Ethyl (*Z*)-2-(2-(2-(benzyloxy)ethyl)-2-methylcyclobutylidene)propanoate.** Triethyl phos-phonopropionate (0.567 g, 2.38 mmol) was added to a suspension of NaH (0.095 g, 2.38 mmol) in THF (10 mL), the mixture was heated to 65 °C and was maintained at that temperature for 6 h. Cyclobutanone (0.519 g, 2.38 mmol) and TDA-1 (0.077 g, 0.24 mmol) in THF (3 mL) was slowly added over 30 min, and the resulting mixture was left heated at 65 °C for 2 days. The reaction was cooled to RT and Et_2_O (50 mL) was added. The ethereal layer was washed with brine (3×50 mL) and dried over Na_2_SO_4_ (anhydr.). The crude product was purified by flash chromatography (Et_2_O in petroleum ether), giving 0.238g of a yellow oil (33% yield, 75% chemical purity according to GC).

^1^H-NMR(CDCl_3_): δ 1.24-1.33 (m, 6H), 1.67-1.72 (m, 4H), 1.94-2.09 (m, 3H), 2.52-2.67 (m, 2H), 3.50-3.56 (m, 5H), 4.15 (q, 2H), 4.48 (m, 2H), 7.32 (m, 5H) ppm. ^13^C-NMR(CDCl_3_): δ 14.44, 25.16, 26.05, 29.22, 37.41, 47.71, 60.08, 68.12, 73.05, 120.30, 127.55, 127.63, 128.45, 138.84, 163.87, 167.19 ppm.

**Ethyl 2-(2-(2-(benzyloxy)ethyl)-2-methylcyclobutyl)propanoate.** Mg(s)(0.153 g, 6.30 mmol) was added to the ethyl ester (0.238 g, 0.79 mmol) in MeOH (2.5 mL) and the reaction was stirred at RT for 29 h. A few drops of HOAc (conc.) was added, and the organic layer was washed with NH_4_Cl (sat.aq., 2×15 mL) and dried over Na_2_SO_4_(anhydr.). Purification by flash chromatography (Et_2_O in petroleum ether) resulted in 0.179 g of a clear oil (74 % yield, 78 % chemical purity according to GC).

^1^H-NMR(CDCl_3_): δ 0.99-1.09 (m, 6H), 1.21-1.26 (m, 3H), 1.64-1.84 (m, 4H), 1.99-2.03 (m, 1H), 2.08-2.13 (m, 2H), 2.45-2.54 (m, 2H), 3.47-3.63 (m, 2H), 4.05-4.13 (m, 2H), 4.48 (m, 1H), 4.51 (s, 1H), 7.28-7.35 (m, 5H) ppm.

**2-(2-(2-(Benzyloxy)ethyl)-2-methylcyclobutyl)propan-1-ol.** LAH (0.022 g, 0.58 mmol) in THF (0.5+0.5 mL) was added dropwise to ethyl ester (0.179 g, 0.58 mmol) in THF (15 mL) at −30 °C. The reaction was quenched after 3h by addition of wet Na_2_SO_4_ (s), and the reaction was filtered over celite. Purification by flash chromatography (Et_2_O in pentane) resulted in 0.173 g clear oil (nearly quantitative yield, 69% chemical purity according to GC).

^1^H-NMR(CDCl_3_): δ 0.86 (s, 3H), 0.87 (s, 3H), 0.88 (s, 3H), 0.89 (s, 3H), 1.07 (s, 3H), 1.08 (s, 3H), 1.08-1.15 (m, 3H), 1.21 (t, 1H), 1.26 (t, 2H), 1.37 (d, 2H), 1.6-1.85 (m, 10H), 1.95-2.02 (m, 1H), 3.26-3.01 (m, 1H), 3.47-3.65 (m, 2H), 3.85-3.88 (m, 1H), 4.48 (s, 1.5H), 4.51 (2H), 5.02 (q, 0.5H), 7.28-7.35 (m, 5H) ppm. Similar to Bernard et al. 2003.

**((2-(2-(1-Iodopropan-2-yl)-1-methylcyclobutyl)ethoxy)methyl)benzene.** Alcohol (0.173 g, 0.66 mmol) in THF (0.5+0.5 mL) was added to imidazole (0.098 g, 1.32 mmol) and TPP (0.346 g, 1.32 mmol) in ACN (4.5 mL). I_2_ (0.335 g, 1.32 mmol) was added to the above mixture after 3 min. After 1h 20 min was the reaction mixture diluted with *n*-pentane (20 mL). The organic layers was washed with NaHSO_3_ (sat.aq., 2 ×10 mL) and dried over Na_2_SO_4_(anhydr.). The obtained crude product was used in the next step without further purification.

**((2-(1-Methyl-2-(prop-1-en-2-yl)cyclobutyl)ethoxy)methyl)benzene.** AgF (0.167g, 1.32 mmol) was added to Iodide (0.245g, 0.66 mmol) in pyridine (2 mL), and the reaction was stirred at RT for 15 h. The reaction mixture was diluted with Et_2_O (50 mL), washed subsequently with CuSO_4_ (sat.aq., 3 × 25 mL) and brine (2 × 25 mL) and dried over MgSO_4_(anhydr.). The crude product was suspended in pentane and stored in the freezer over night. Filtration and evaporation resulted in 0.108 g yellow oil/white crystals. Purification with flash chromatography (Et_2_O in *n*-pentane) resulted in 0.089 g clear oil in a 1:0.8 ratio of two isomers (87 % chemical purity, 55 % yield over two steps).

^1^H-NMR(CDCl_3_): δ 0.91 (s, 3H), 1.15 (s, 3H), 1.64 (s, 1.5H), 1.65 (s, 1.5H), 1.77-1.86 (m, 4H), 1.93-2.01 (m, 1H), 2.52-2.58 (m, 1H), 3.46-3.56 (m, 2H), 4.49 (s, 1H), 4.49 (s, 1H), 4.61 (s, 0.5H), 4.63 (s, 0.5H), 4.81 (bs, 1H), 7.33 (m, 5H) ppm. Similar to Bernard et al 2003.

***rac*-Grandisol and *rac*-fragranol.** To a dark blue solution of Li (approx. 5 mg, 4.8 mmol) in liquid NH_3_ (ca. 10 mL) at −78 °C was added under argon a 1:0.8 mixture of the *cis-* and *trans-*isomers of ((2-(1-methyl-2-(prop-1-en-2-yl)cyclobutyl)ethoxy)methyl)benzene (45 mg, 0.182 mmol) in Et_2_O (0.5 mL) all at once. The mixture was stirred for 35 min and quenched rapidly with MeOH (10 mL). The solution was allowed to reach room temperature overnight, treated with saturated aqueous NH_4_Cl and extracted with Et_2_O. The extract was dried over Na_2_SO_4_(anhydr.) and concentrated in vacuum affording approximately 15 mg (58 % yield) of a 1:0.8 mixture of racemic grandisol and racemic fragranol. SPE column purification resulted in fractions containing pure grandisol (fraction 8-9), and a mixture (fractions 10-12) and then fractions mainly containing fragranol (fractions 13-15).

^1^H-NMR(CDCl_3_): δ 0.94 (s, 3H, fragranol), 1.17 (s, 2H, grandisol), 1.11 (bs), 1,41-1.47(m, 2H), 1.58-1.67 (m, 0.7H), 1.67 (s, 3H, fragranol), 1.69 (s, 2H, grandisol), 1.73-1.88 (m, 5H), 1.93-2.03 (m, 2H), 2.55 (t, 0.6H, grandisol), 2.58 (t, 1H, fragranol), 3.63-3.74 (m, 3.6H), 4.62 (s, 1H), 4.65 (s, 0.7H), 4.89 (s, 1.7H) ppm. NMR was run on the SPE fractions 10-12 containing all four stereoisomers.

**Protocol for enantiomeric enrichment of (−)-(*R*)-terpinen-4-ol**

**3,5-Dinitrobenzoyl derivative of (−)-(*R*)-terpinen-4-ol.** *n*-BuLi [85 mL, 136 mmol (1.6 M in *n*-hexane)] was added drop wise (30 min) to a solution of (−)-(*R*)-terpinen-4-ol [20.0 g, 130 mmol (*S*:*R* = 24.5:75.5)] in THF (200 mL) cooled on an ice bath under an argon atmosphere. 3,5-Dinitrobenzoyl chloride (33.0 g, 143 mmol) was then added portion wise (5 min), and the reaction mixture was stirred at room temperature. The color went from deep red to an orange turbid mixture. After four hours, aqueous saturated NaHCO_3_ (100 mL) was added to the reaction mixture, and the organic layer was removed, followed by extraction of the aqueous layer with EtOAc (4 x 50 mL). Ddrying of the combined extracts over MgSO_4_ and evaporation of the solvent at reduced pressure resulted in a light orange solid, which was purified by flash chromatography (Silica, EtOAc/*n*-hexane) to remove residues of unreacted alcohol. 41.9 g (120 mmol, 93% yield, yellow/orange crystals). ^1^H NMR (500 MHz, CDCl_3_): δ 0.98 (3H, d, *J* = 7.2 Hz), 0.99 (3H, d, *J* = 7.2 Hz), 1.66 (3H, s), 1.81-1.88 (1H, m), 1.95-2.06 (2H, m), 2.32-2.38 (1H, dm), 2.56-2.63 (2H, m), 2.89 (1H, heptet, *J* = 6.9 Hz), 5.35 (1H, bs), 9.04 (2H, d, *J* = 2.2 Hz), 9.19 (1H, t, *J* = 2.2 Hz); ^13^C NMR (62.5 MHz, CDCl_3_): δ 17.24, 17.66, 23.09, 27.47, 27.74, 29.93, 30.80, 90.41, 117.36, 121.94, 129.17, 133.59, 135.73, 148.62, 161.41.

**Recrystallisation**. The dinitrobenzoate (41.9 g, *S*:*R* = 24.5:75.5) was dissolved in boiling 99.5% ethanol (1000 mL) and was allowed to cool slowly and crystallise overnight. The crystals (30 g, *S*:*R* = 30.8:69.2) were redissolved in boiling *iso*-propanol (1000 mL) and the solution was allowed to cool slowly and crystallize for three days. In both recrystallisations, the *R*-isomer was enriched in the solution. The crystals were filtered off and the solvent was evaporated, resulting in bright yellow/beige crystals (4.9 g, *S*:*R* = 2.4:97.6) from the isopropanol solution and (11.6 g, *S*:*R* = 7.5:92.5) from the ethanol solution. The enantioenriched crystals were combined (16.5 g, *S*:*R* = 3.2:96.8) and dissolved in boiling *iso*-propanol (1000 mL). The solution was allowed to cool slowly and crystallize for eight days. This time, the *R*-isomer was enriched in the precipitated crystals (1.67 g, *S*:*R* = 0.3:99.7). The solution was allowed to stand for another six days (8 °C), which resulted in additional crystals (4.95 g, *S*:*R* = 0.5:99.5)

The *S*:*R*-ratios were determined by GC on the alcohols on a β-dex 225 capillary column (30 m x 0.25 mm x 0.25 μm, 100 °C isothermal, He: 12 psi), by hydrolysing a “GC-sample” with 2.4 M KOH in 10% water in MeOH.

**Hydrolysis of 3,5-dinitrobenzoyl derivative.** 2.4 M KOH in 10% water in MeOH (40 mL) was added to the 3,5-dinitrobenzoate (1.63 g, 4.68 mmol, *S*:*R* = 0.3:99.7). The mixture was refluxed for 10 min and then stirred over night at room temperature. Water (50 mL) was added followed by extraction with *n*-pentane (4 x 25 mL) and drying of the combined extracts over MgSO_4_. Evaporation of solvent at reduced pressure resulted in an almost colorless oil, which was purified by bulb-to-bulb distillation (90 °C/2.9 mbar). The enantioenriched (−)-(*R*)-Terpinen-4-ol was obtained as a colorless oil, 700 mg (4.54 mmol, 97% yield, 98.5% purity, *S*:*R* = 0.4:99.6). [α]_D_^20^ -52.2 (c 1.10, CHCl_3_); ^1^H NMR (500 MHz, CDCl_3_): δ 0.93 (3H, d, *J* = 6.9 Hz), 0.95 (3H, d, *J* = 6.9 Hz), 1.46 (1H, s), 1.53-1.59 (1H, m), 1.63-1.71 (5H, m), 1.89-1.96 (2H, m), 2.12-2.20 (2H, m), 5.30 (1H, bs); ^13^C NMR (62.5 MHz, CDCl_3_): δ 16.82, 16.84, 23.28, 27.05, 30.77, 34.61, 36.77, 71.76, 118.43, 133.88.

References

Bernard AM, Frongia A, Secci F, Delogu G, Ollivier J, Piras PP, Salaun J (2003) Stereospecific palladium(0)-catalyzed reduction of 2-cyclobutylidenepropyl esters. A versatile preparation of diastereomeric monoterpenoids: (±)-fragranol and (±)-grandisol. Tetrahedron 59:9433–9440. [https://doi.org/10.1016/j.tet.2003.09.074](https://doi.org/10.1016/j.tet.2003.09.074)
